# Expression of regulatory receptors on γδ T Cells and their cytokine production in Behcet's disease

**DOI:** 10.1186/ar4147

**Published:** 2013-01-21

**Authors:** Gunes Parlakgul, Ekin Guney, Burak Erer, Zeki Kılıcaslan, Haner Direskeneli, Ahmet Gul, Guher Saruhan-Direskeneli

**Affiliations:** 1 Istanbul Medical Faculty, Istanbul University, Millet Caddesi, Çapa, Istanbul, 34093 Turkey; 2 Division of Rheumatology, Department of Internal Medicine, Istanbul Medical Faculty, Istanbul University, Millet Caddesi, Çapa, Istanbul, 34093 Turkey; 3 Department of Pulmonary Diseases, Istanbul Medical Faculty, Istanbul University, Millet Caddesi, Çapa, Istanbul, 34093 Turkey; 4 Division of Rheumatology, Department of Internal Medicine, Marmara University, School of Medicine Hospital, Fevzi Çakmak Mahallesi, Mimar Sinan Caddesi, No: 41, Pendik, Istanbul, 34890 Turkey; 5 Department of Physiology, Istanbul Medical Faculty, Istanbul University, Millet Caddesi, Çapa, Istanbul, 34093 Turkey

## Abstract

**Introduction:**

Behcet's disease (BD) is a multi-systemic disorder with muco-cutaneous, ocular, arthritic, vascular or central nervous system involvement. The role of γδ T cells is implicated in BD. The activation status of γδ T cells and their cytokine secretion against phosphoantigens are evaluated in BD.

**Methods:**

NKG2A, NKG2C, NKG2D, CD16 and CCR7 molecules on γδ T cells were analyzed in 70 BD, 27 tuberculosis (TB) patients and 26 healthy controls (HC). Peripheral γδ T cells were expanded with a phosphoantigen (BrHPP) and IL-2, restimulated with BrHPP and a TLR3 ligand, and cytokine production was measured.

**Results:**

γδ T cells were not increased in both BD and TB patients, but the proportions of TCRVδ2^+ ^T cells were lower (58.9 and 50.7 vs. 71.7%, *P *= 0.04 and *P *= 0.005) compared to HC. Higher proportion of TCRVδ2^+ ^T cells were CD16^+ ^(26.2 and 33.9 vs. 16.6%, *P *= 0.02 and *P *= 0.001) and CCR7^- ^(32.2 and 27.9 vs. 17.7%, *P *< 0.0001 and *P *= 0.014) in BD and TB patients compared to HC. NKG2C^+ ^γδ^+ ^T cells were relatively increased (0.5 and 0.6 vs. 0.3%, *P *= 0.008 and 0.018), whereas NKG2D positivity was decreased in patients with BD and TB (77.7 and 75.8 vs. 87.5%, *P *= 0.001 and 0.004). Expansion capacity of γδ T cells in BD and TB as well as production of IL-13, IFN-γ, granulocyte monocyte colony stimulating factor (GM-CSF), TNF-α, CCL4 and CCL5 in BD was lower compared to HC, when restimulated by TLR3 ligand and BrHPP.

**Conclusion:**

The changes on γδ T cells of BD as well as TB patients implicate that γδ T cells have already been exposed to regulatory effects, which changed their activity. Lower cytokine response of γδ T cells implicates down modulation of these cells in BD.

## Introduction

Behcet's disease (BD) is a systemic inflammatory disorder with a diverse spectrum of clinical manifestations including mucocutaneous, ocular, vascular, gastro-intestinal, musculoskeletal and central nervous system involvements. A complex genetic background leading to a pro-inflammatory, innate immune system derived activation perpetuated by adaptive immune responses against environmental or auto-antigens is considered as the pathogenic mechanism in BD [1-3].

γδ T cells represent a minor T cell population (1 to 10% of peripheral blood (PB) T cells) with combining properties of adaptive and innate immunity that express T cell receptors (TCRs) comprised of γ and δ heterodimer. TCRVγ9Vδ2^+ ^T cells, the major subset of γδ T cells (70 to 95%) in the PB in humans, recognize non-peptidic phosphoantigens produced by microbial and eukaryotic mevalonate pathways in a TCR-dependent manner [4]. In adults with negative purified protein derivative (PPD) response, TCRVγ9Vδ2^+ ^T cells reacting to isopentenyl pyrophosphate (IPP) secrete Th1 type cytokines (IFN-γ, TNF-α) and MIP-1β (CCL4) [5]. TCRVγ9Vδ2^+ ^T cells stimulated in the presence of growth factors and cytokines can produce abundant amounts of the pro-inflammatory cytokines and change their phenotype from memory cells expressing CCR7^+ ^to CCR5^+ ^expressing cells [6].

The low affinity immunoglobulin receptor (FcγRIII) CD16 is also shown to discriminate between two subsets of TCRVγ9Vδ2^+ ^T cells with distinct functional responses [7]. The expression of CD16 regulated cytolytic activity and production of inflammatory cytokines of the γδ T cells.

γδ T cells also express inhibitory and activating members of the NK receptor family and modulate their effector functions, such as cytotoxicity and cytokine production. Among the NK receptors, heterodimeric "killer lectin receptors" of CD94 with NKG2A or NKG2C, exerting inhibitory or activating effects, respectively, interact with the nonclassical MHC class Ib molecule HLA-E [8]. The majority of TCRVγ9Vδ2^+ ^T cells harbor inhibitory receptors with CD94/NKG2A heterodimers and these cells exhibit a strong lytic activity [9].

Due to their small number in the peripheral blood, human γδ T cells are often expanded before functional studies are performed. Short-term γδ T cells can be generated from PB by stimulating with γδ T cell specific phosphoantigens and IL-2 *in vitro*. Depending on the stimulus and IL-2, purity of these cells may reach up to 70 to 95% [10]. As these expanded human γδ T cells are shown to express mRNA of TLR1-10 receptors similar to freshly isolated γδ T cells, costimulatory effects of TLR ligands could be demonstrated *in vitro *[11,12].

Peripheral blood γδ T cells were elevated in BD, with a polyclonal activation [13,14]. γδ T cells were associated with active BD with higher expression of CD69 and production of IFN-γ and TNF-α [15]. IPP-specific TCRVγ9Vδ2^+ ^Th1-like cells from intra-ocular fluid are generated from the eye of BD patients with uveitis [16]. In Italian BD patients, TCRVγ9δ2^+ ^T lymphocytes were expanded and shown to express TNF receptor II and IL-12 receptor β1 in active disease [17].

A more prominent pro-inflammatory cytokine secretion was shown from peripheral blood mononuclear cells (PBMCs) in BD patients in response to various antigens, including IPP and PPD, and increased IFN-γ and IL-12 secretion was detected [18]. In this study, *ex vivo *PBMC are studied to evaluate γδ T cells as a candidate for an immune effector cell group in BD, in tuberculosis (TB) as an infectious disease control and in healthy controls (HC).

## Materials and methods

### Patients

The patient group consisted of 70 patients with Behçet's disease (48 men and 22 women, mean age 36.8 ± 11.0 years), followed at the BD Outpatient Clinic of the Division of Rheumatology, Istanbul Faculty of Medicine, Istanbul University. All patients met the International Study Group criteria for the classification of BD [19]. Active disease was defined as the presence of ongoing disease activity depending on the assessment of any exacerbation of characteristic BD symptoms within the last four weeks or at the first visit, including severe skin, mucosal and/or ocular manifestations that required introduction or increase of systemic corticosteroids (Table 1). At the time of blood sampling, disease was active in 38 patients. Of the active patients, 36 were using colchicine, 24 of them were using immunosuppressant agents, such as azathioprine (*n *= 13) or corticosteroids (*n *= 19), and 2 patients were untreated. Seven inactive patients were using only colchicine and 25 patients were on colchicine as well as an immunosuppressant agent, such as azathioprine (*n *= 13) and low or medium dose corticosteroids (*n *= 14). Twenty-seven active TB patients (20 men and 7 women, mean age 37.0 ± 15.7 years) and 26 healthy volunteers (HC, 22 men and 4 women, mean age 38.1 ± 9.5 years) were enrolled as control groups. Ethics Committee of Istanbul Faculty of Medicine approved the study protocol, and all study donors provided written informed consent according to the Declaration of Helsinki prior to blood collection.

**Table 1:** Clinical features of 70 BD patients with active (A-BD) and inactive disease (IA-BD)

Clinical manifestations	A-BD patients N (%)	IA-BD patients N (%)
	38	32
Oral ulcer	22 (57.9)	23 (71.9)
Genital ulcer	13 (34.2)	9 (28.1)
Skin manifestations	21 (55.3)	24 (75)
Pathergy	8 (21.1)	15 (46.9)
Joint manifestations	14 (36.8)	7 (21.9)
Uveitis	6 (15.8)	4 (12.5)
Vascular manifestations	11 (28.9)	7 (21.9)

### Cell preparation - flow cytometry

PBMCs were isolated from EDTA- anticoagulated venous blood by density-gradient centrifugation (Lymphoprep, Eurochrom, Berlin, Germany). Fluorescence-conjugated monoclonal antibodies (mAb) and their target antigens used in the study were as follows: CD45/FITC (fluorescein isothiocyanate)-CD14/R-PE (R-phycoerythrin) (Ancell Corporation, Bayport, MN, USA), CD16/PC5 (anti-FcγRIII, 368), CD56/PC5 (anti-NCAM, N901), pan-γδ/FITC (Immu510), δ2/FITC (Immu389), CD8/PC5 (B9.11), NKG2D/PE (ON72), NKG2A/PE (Z199, Beckman-Coulter, Immunotech, Marseille Cedex, France), CD94/FITC (70 kDa dimer, Kp43, HP-3D9, BD Biosciences, USA), NKG2C/PE (134591) and CCR7/PE (150503, R&D Systems, USA). For every antibody combination, 1 μl of antibody per 10^5 ^PBMCs was incubated at 4°C for 20 minutes, washed and analyzed by flow cytometer (Epics XL, Beckman-Coulter, USA). Results are expressed as percentages of the respective positive cells in the lymphocyte gate.

### *In vitro *expansion of TCRVγ9Vδ2^+ ^T lymphocytes

PBMCs were cultured in 96-well plates at a concentration of 10^6 ^cells/ml in RPMI 1640 supplemented with 10% fetal calf serum (Gibco, Life Technologies, USA), 2 mM L-glutamine, 1% sodium pyruvate and 100 U/ml penicillin/streptomycin (Sigma, Deisenheim, Germany) at 37°C and 5% CO_2_. The proportion of γδ T cells was measured on the first day. For the expansion of TCRVγ9Vδ2^+ ^T cells, PBMCs were cultured in medium alone or in the presence of a synthetic analogue of natural phosphoantigens (bromohydrin pyrophosphate, BrHPP, 3 mM, kindly provided by Helene Sicard, Innate Pharma, Marseille, France). Responsive γδ T cells were expanded by adding 100 IU/ml recombinant human interleukin-2 (IL-2) on the second and fifth day of the cell culture. After eight days, cells were washed and expansion of TCRVγ9Vδ2^+ ^T cells was assessed by staining the cultured cells with anti-TCRVδ2 (Immu389), as described above. Cultures of donors with at least 70% γδ^+ ^(TCRVδ2^+^) T cells (23 active BD, 20 inactive BD, 9 TB, 20 HC) were restimulated with the 50 mg/ml Polyinosinic:polycytidylic acid (PolyI:C, TLR3 agonist), 3 mM BrHPP or PolyI:C and BrHPP at the eighth day. The levels of IL-13, interferon-γ (IFN-γ), tumor necrosis factor-α (TNF-α), granulocyte monocyte colony stimulating factor (GM-CSF), CCL4 (macrophage inflammatory protein 1-β) and CCL5 (RANTES, Regulated on Activation, Normal T Expressed and Secreted) were measured in the supernatants after 24 hours of the re-stimulation by multiplex ELISA (Procarta, Luminex, Affymetrix, CA, USA).

### Statistical analysis

The results are presented as mean and standard deviations throughout the manuscript. Cytokine levels were measured as "median intensity values", and the direct readings were evaluated instead of transformation to pg/ml. The parameters compared between patients and controls were subjected to the non-parametric Mann-Whitney U-test. The proportions of expanded γδ T cells were compared with the chi square test.

## Results

### Phenotype of γδ T lymphocytes in peripheral blood

The proportions of γδ^+ ^T cells in PBMC were relatively low in BD patients and in both disease (TB) and healthy control (HC) groups (Table 2). Pan γδ^+^T cells were slightly increased only in patients with inactive BD (IA-BD) compared to HC (Mean: 4.7 vs. 3.6%, *P *= 0.029). The difference of pan γδ^+^T cells between active BD (A-BD) and IA-BD patients was not significant. The proportion of major γδ T cell population in the PB, namely TCRVγ9Vδ2^+ ^T cells (shown with anti-Vδ2 antibody) was similar in all groups (Table 2). When compared among the pan γδ^+ ^T cells, the dominance of TCRVδ2^+ ^T cells was less prominent in BD and particularly in the IA-BD group compared to HC (58.9 and 54 vs. 71.7%, *P *= 0.04 and *P *= 0.014, respectively). This significantly lower proportion of TCRVδ2^+ ^of pan γδ^+ ^T cells was observed in TB as well (50.7%, compared to HC *P *= 0.005).

**Table 2:** Phenotypic features of γδ T cells in BD, TB patients and healthy controls (HC)

Subtype	BD	TB	HC	BD vs. HC	TB vs. HC
	**70**	**27**	**26**		
**γδ^+ ^T**	4.3 ± 2.2	3.5 ± 2.6	3.6 ± 2.5		
**TCRVδ2^+ ^T**	2.6 ± 1.9	2.2 ± 2.5	2.4 ± 2		
**TCRVδ2^+ ^T of γδ T**	**58.9 ± 24.3**	**50.7 ± 23.8**	71.7 ± 25.7	*0.04*	*0.005*
**CD16^+ ^of TCRVδ2^+ ^T**	**26.2 ± 19.6**	**33.9 ± 19.1**	16.6 ± 13	*0.02*	*0.001*
**CCR7^- ^of TCRVδ2^+ ^T**	**32.2 ± 18.5**	**27.9 ± 15.9**	17.7 ± 9.2	*< 0.001*	*0.014*
**NKG2A^+ ^γδ^+ ^T**	1.8 ± 1.4	1.3 ± 1.6	1.2 ± 0.7		
**NKG2A^+ ^of γδ^+ ^T**	38 ± 20.9	32.6 ± 20.3	39.4 ± 15		
**NKG2C^+ ^γδ^+ ^T**	**0.53 ± 0.44**	**0.64 ± 0.8**	0.3 ± 0.3	*0.008*	*0.018*
**NKG2C^+ ^of γδ T**	13.5 ± 11.8	14.3 ± 13.9	11.3 ± 9.3		
**NKG2D^+ ^γδ^+ ^T**	3.4 ± 1.9	2.7 ± 2	2.8 ± 1.4		
**NKG2D^+ ^of γδ T**	**77.7 ± 15.3**	**75.8 ± 17.5**	87.5 ± 11.5	*0.001*	*0.004*

Mean values ± S.D. are presented. *P*-values of respective comparisons are listed in left columns.

**Figure 1:**
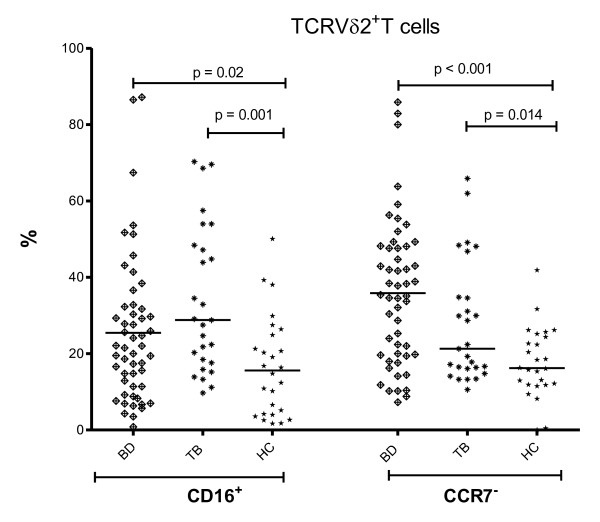
**CD16^+ ^or CCR7^-^TCRVδ2^+ ^T cells**. The proportion of TCRVδ2^+ ^T cells carrying CD16 or lacking CCR7 in the peripheral blood of 70 BD (38 A-BD, 32 IA-BD), 27 TB patients and 30 HC. The differences of BD and TB with HC were significant (*P *= 0.02 and *P *= 0.001, and *P *< 0.001 and *P *= 0.014, respectively).

To access the functional status of the γδ T cells, surface expression of several co-stimulatory receptors was screened on these cells. The immunoglobulin receptor CD16, discriminating two subsets of TCRVγ9Vδ2^+ ^T cells with distinct functional responses, was evaluated as [7] an activation receptor for TCRVδ2^+ ^T cells. CD16 expression on these cells was higher in both in BD patients (including active and inactive) compared to HC (26.2 vs. 16.6%, *P *= 0.02). This TCRVδ2^+ ^T cell population expressing CD16 was even more increased in TB group compared to HC (33.9%, *P *= 0.001, Figure 1).

When pan γδ^+ ^T cells are analyzed for expression of NKG2A or NKG2C receptors, the higher fraction of TCRVγ9Vδ2^+ ^T cells expressed inhibitory NKG2A rather than activating NKG2C in all groups as shown previously [9] (Table 2). Similar to a previous study [20], the fraction of NKG2C^+ ^γδ^+ ^T cells varied widely in all groups of this study. However, a slight increase of activating NKG2C^+^γδ^+ ^T cells among the PB lymphocytes was observed in the BD group compared to the HC (0.5 vs. 0.3%, *P *= 0.008). Namely, the NKG2C^+ ^fraction of pan γδ^+ ^T cells was increased in BD compared to controls without reaching statistical significance (13.5 vs. 11.3%). The relative increase of NKG2C^+ ^γδ^+^T cells was also detected in TB (0.6% of PB lymphocytes, *P *= 0.018, and 14.3% of γδ^+ ^T cells, Table 2).

**Figure 2:**
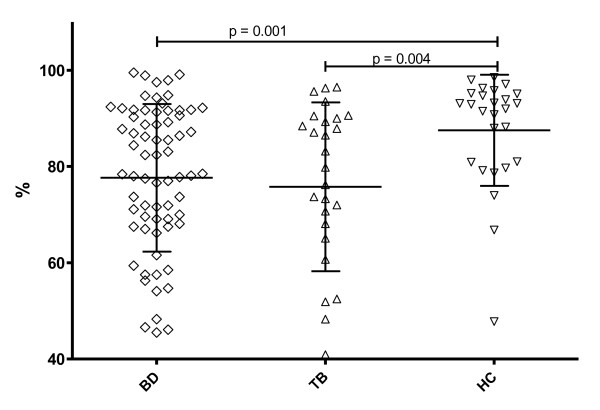
**TCR γδ^+ ^T cells carrying NKG2D**. The % of TCR γδ^+ ^T cells carrying NKG2D in the peripheral blood of 70 BD (38 A-BD, 32 IA-BD), 27 TB patients and 30 HC. The differences between BD and TB to HC were significant (*P *= 0.001 and *P *= 0.004).

TCRVγ9Vδ2^+ ^T cells can also be activated through NKG2D independently of antigen [21] or by ligands of NKG2D, that is, MICA or MICB, which are often induced by stress [22]. NKG2D receptors on the γδ T cells, however, were decreased in patients with BD and TB compared to HC (77.7 and 75.8 vs. 87.5%, *P *= 0.001 and 0.004, Figure 2).

CCR7 is used to distinguish tissue-homing effector and lymph node-homing non-effector memory cells [23]. When expression of CCR7 was investigated, CCR7^- ^of TCRVδ2^+ ^cells were increased in BD compared to HC (32.2 vs. 17.7%, *P *< 0.001) implicating an increase of effector γδ T cell phenotype in the periphery by losing their CCR7 [24]. These CCR7^- ^γδ T cells were also more frequent in PB of TB patients compared to HC (27.9%, *P *= 0.014, Figure 1).

These data showed, despite the comparable number of γδ T cells in BD as in HC, that increased proportions of CD16^+^, NKG2C^+ ^or CCR7^- ^γδ^+ ^T cells with an effector phenotype were evident in BD as well as in TB.

### *In vitro *cytokine and chemokine responses of short-term γδ^+ ^T cell lines

**Figure 3:**
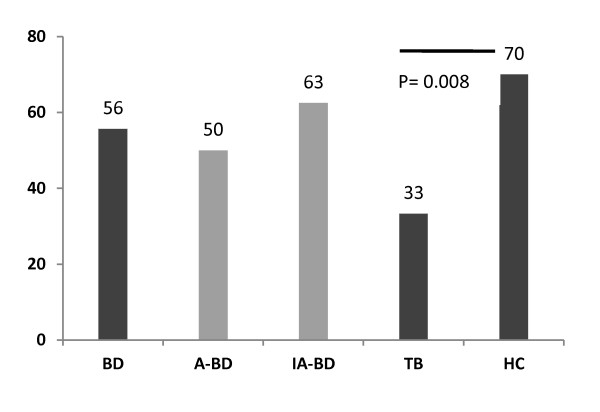
**Ratio of expanded γδ^+ ^T cells**. The % ratio of γδ^+ ^T cells expanded over 70% in 70 total BD (in A-BD and IA-BD), 27 TB patients and 30 HC. The difference between TB and HC groups was significant (*P *= 0.008).

To evaluate the effector functions, such as *in vitro *expansion capability and cytokine activity of γδ T cells in BD, phosphoantigen analog BrHPP was used to stimulate these cells and responsive cells were propagated with IL-2. From the γδ T cell cultures of 70 BD and 27 TB patients and 30 HC donors, 55.7% (50% in A-BD and 62.5% in IA-BD), 33.3% and 70% could be expanded up to > 70% TCRVδ2^+ ^at the eighth day *in vitro*, respectively. The mean expansion ratio of BD patients was lower in A-BD than IA-BD and in HC without statistical significance. However, this expandability of cells was significantly lower in TB patients compared to HC (*P *= 0.008) (Figure 3). The expanded γδ T cells were used further *in vitro *to compare their responsiveness to stimuli by cytokine secretion.

When stimulated with phosphoantigens via TCR, various cytokine and chemokine production can be induced in γδ T cells [25]. Phosphoantigen reactive γδ T cells represented a type-1 polarized subset secreting IFN-γ and TNF-α [26]. Expanded γδ T cells of patients and controls containing over 70% γδ T cells (short-term γδ T cell lines) were restimulated by TCR and compared for cytokine and chemokine secretions in culture supernatants from 42 BD, 9 TB patients and 20 HCs. The phosphoantigen re-stimulation induced in these short-term γδ T cell lines to produce higher levels of IFN-γ, GM-CSF, IL-13, TNF-α as well as CCL4 (MIP1-β) and CCL5 (RANTES) in all patient and control groups (Additional file 1, Figure S1 shows the results in HC). Induction of IFN-γ and IL-13 production of short-term γδ T cell lines by TCR (BrHPP) re-stimulation did not differ among groups, whereas GM-CSF, TNF-α, CCL4 and CCL5 production was lower in BD compared to HC (*P *= 0.02, 0.015, 0.05, respectively).

**Figure 4:**
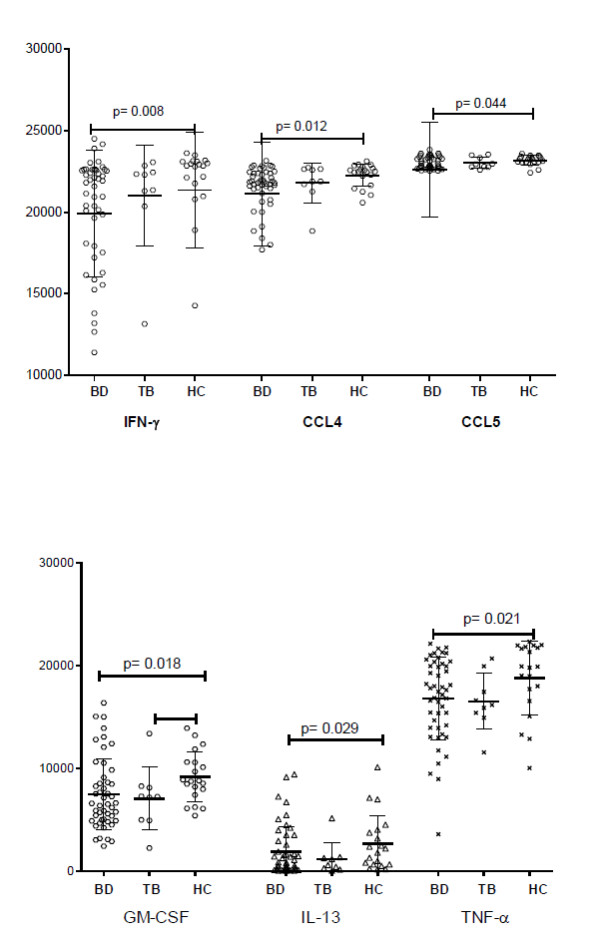
**Production of cytokines with TCR and co-stimulation of TLR3 of γδ T cells**. IFN-γ, CCL4 and CCL5 as well as GM-CSF, IL-13 and TNF-α levels with TCR and co-stimulation of TLR3 were significantly lower in BD, including active and inactive patients, than in HC (*P *= 0.008, 0.012 and 0.044 and 0.018, 0.029 and 0.021, respectively).

An interaction of TCR- and TLR-signaling is shown to enhance effector functions of human PB γδ T cells. To investigate the activating or co-stimulating properties of TLR3 on γδ T cells, a TLR3 ligand was added to the cells at the re-stimulation stage to mimic the responses to specific and non-specific stimulations. The re-stimulation with TCR and TLR3 agonist simultaneously induced significantly lower levels of IFN-γ, IL-13, TNF-α, as well as GM-CSF, CCL4 and CCL5, in BD than in HC (*P *= 0.008, 0.029 and 0.021, 0.018, 0.012 and 0.044, respectively) (Figure 4).

As 42% of the patients were on immunosuppressive treatment at the time of blood sampling, we compared the patients with or without treatment to see the possible effect of treatment. The responses to TCR stimulation did not show a significant difference between the groups receiving a treatment or not.

## Discussion

γδ T cells have been evaluated in BD in various studies with a possible role in bridging the innate and adaptive immune activations and functioning as a local defense response inducing activation of other immune cell populations. Data such as tissue infiltrations of γδT cells in erythema nodosum-like lesions of BD patients suggested a role for γδ T cells in disease pathogenesis. In this study, γδ T cells are evaluated in BD and as a disease control group in TB for their activation state and for their functional activities in response to stimuli.

When the presence of this rare cell population in BD is compared to healthy individuals, conflicting results have been published with [13,14,27] and without [16,28] increased levels in the PB, with a predominantly disease exacerbation-related increase in recent studies [14,17]. In the present study, which investigated a relatively higher number of BD patients, γδ T cells were only slightly increased in the blood of the patients.

The discrepancy among the previous studies about the number of γδT cells in PB might be due to the activation status of the disease, as reflection of local tissue inflammation to PB might be variable due to factors such as the disease severity or immunosuppressive medications. Even acute-phase response (erythrocyte sedimentation rate (ESR), C-reactive protein (CRP)), which is the hallmark of inflammation, is not a reliable biomarker in BD and elevated only in relation to a limited number of disease manifestations, such as arthritis or some skin lesions of the disease. Similar to our results, in a previous study, although lesional skin of patients with BD had significantly increased γδ T cells, peripheral blood γδ T cells counts were similar in BD and control groups [29].

Despite the dominance of the TCRVδ2^+ ^subtype among the γδ T cells in BD patients similar to previous reports [30], we found a decrease of TCRVδ2^+ ^proportion of γδ T cells in BD compared to HC. Considering γδ T cells as an effector cell type against mycobacterial antigens, TB patients had also relatively low γδ T cells and lower proportions of TCRVδ2^+ ^T cells among pan γδ T cells.

In BD, PB γδ T cells expressed early activation markers and secreted Th1 and proinflammatory cytokines. Bacteria-specific KTH-1 responsive γδ T cell lines have been demonstrated to be pro-inflammatory, as well. Significantly greater proportions of the Vδ1^+ ^and Vδ2^+ ^γδ T cell subsets were activated in patients with active BD; however, the balance of activation favored Vδ1^+ ^T cells [31]. In this present study, we have not evaluated Vδ1^+ ^T cells separately; however, the total number of the γδ T cells was not increased, suggesting their relatively low increase, if any, as well. As γδ T cells are more likely to be tissue resident and relatively rare in the blood, the phenotypic differences observed between studies may be related to wide distribution features of cells in systemic circulation and tissues.

Based on previous data of the activated status of γδ T cells in BD [17], when phenotypic markers for effector cells were evaluated, the data revealed an increased activation status of the γδ T cells in BD and also in TB. The elevation of FcγRIII (CD16) expression on γδ T cells was in accordance with some autoimmune diseases, such as Sjogren's syndrome or MS [32,33]. Similarly, effector or central memory T subtypes are proposed for γδ T cells based on their CCR7 expression [34]. γδ T cell activation results in an immediate acquisition of secondary lymphoid tissue-homing capabilities, including CCR7 expression, which decline afterwards [35]. A relative increase of γδ T cells without the lymphoid homing receptor emphasizes effector features of these cells based on not a recent activation in BD as well as in TB compared to HC. IFN-γ producing γδ T cells in response to mycobacterium antigen also had mostly an effector memory cell phenotype of CD45RA^- ^CCR7^- ^in a recent study [36].

γδ T cells appear to play a predominant role against mycobacterium tuberculosis infection and produce IFN-γ strongly and early in response to mycobacterial phosphoantigens [37,38]. These cells, however, are quantitatively and functionally impaired by mycobacterial infection; the percentage of the Vδ2 subset was found to be lower in patients with TB than in controls [39] creating an imbalance between the Vδ1 and the normally predominant Vδ2^+ ^subsets and Vδ2^+ ^anergy [40]. Reduced circulating Vγ9Vδ2^+ ^T cell effectors in patients with acute pulmonary tuberculosis resulted in a reduced frequency of IFN-γ-producing cells after stimulation with nonpeptidic mycobacterial ligands as well [41]. TCRVδ2^+ ^T cells in BD resemble these cells with down-regulated status in PB.

γδ T cell effector function is also modulated by inhibitory and activating NK receptors. A large fraction of circulating Vγ9Vδ2 T cells express MHC class I receptors, mostly the inhibitory CD94/NKG2A complex. Triggering of this receptor down-modulates activation of the γδ TCR and inhibits γδ T cell proliferation and IFN-γ and TNF-α production in response to microbial phosphorylated ligands representing a mechanism to control self-reactivity of γδ T cells. Following stimulation with mycobacterial ligands, the CD94/NKG2A complex has been shown to rapidly translocate to the cell membrane on Vδ2^+ ^cells, leading to inhibition of cell activation [42]. In the present data from BD and TB patients, inhibitory regulation of Vδ2^+ ^T cells was not up-regulated, but the small number of activating receptor NKG2C^+ ^γδ T cells were relatively increased, implicating not a recent activation inducing NKG2A on δ2^+ ^T cells.

Human Vδ2^+ ^subset of γδ T cells can co-express both activating and inhibitory NK receptors with FcγRIII (CD16). CD94/NKG2C complex is functional with IFN-γ production, T cell proliferation and cytolytic activity, whereas NKG2A expression was found on all γδ T cell memory subsets, suggesting a crucial role of the inhibitory signal provided by this receptor on γδ T cell responses [20]. In BD and in A-BD a slight increase of activating NKG2C^+^γδ T cells has been detected showing an activation status of the cells, although the other activating receptor, NKG2D, was decreased in BD. Both of these changes were also detected in the TB group, suggesting similar functional changes of γδ T cells in both diseases.

Functional alterations in BD were evident as the expansion ratio of γδ T cells was significantly lower in BD and more so in TB, implicating an unresponsiveness of these cells for re-stimulation *in vitro*. That was contrary to the previous data on BD, as the expansion factor of γδ T cells was greater in patients with active disease than in those with inactive disease or in control individuals [17], which we did not replicate.

Vδ2^+ ^T cells have been shown to regulate other cells by secretion of cytokines and chemokines, such as CCL4 and CCL5 [6,25], in response to components of bacterial cell walls, supporting a role for these cells in the early stages of the inflammatory responses to many common pathogens. However, the GM-CSF, CCL4, CCL5 and TNF-α responses of the expanded cells to TCR stimulation was lower in BD, especially in IA-BD patients as in TB. This low response could be the result of the *in vitro *expansion step, which may have caused an anergy in these cells. But also an *in vivo *exhaustion of γδ T cells could not be ruled out, although the expandability of the cells points to their capability to respond to antigenic stimulation *in vitro *[40].

Although TLR3 is not expressed on the cell surface of freshly isolated γδ T cells, the co-stimulatory effect of TLR3 ligation has been shown and it is up-regulated after TCR-stimulation and increased IFN-γ secretion [12]. We assumed that activation and expansion of γδ T cells before co-stimulation with TLR3 agonists, could reveal the effect of TLR3 ligand at the re-stimulation in our system. However, the results showed that the short-term T cell lines of BD patients responded with less cytokine secretion to TCR and TLR than HC. Similar to our results, the response of expanded γδ T cells to TLR ligand co-stimulation has been shown to be somehow lower than TCR/TLR3 ligand stimulated freshly isolated γδ T cells [10]. This effect was even more pronounced in BD patients.

The functional data presented are derived from PB cells expanded *in vitro *before testing. Due to the low number of γδ T cells in PB, *in vitro *manipulation and stimulation has been undertaken. However, with the expansion cut-off, we also had to measure the activity of 30% non-γδ T cells, which could not be avoided and limits the interpretation of this functional data.

The results only represent a subset of γδ T cells as mainly Vγ9Vδ2 T cells are responding to phosphoantigens and expand in culture. This subgroup has become the most widely studied γδ T cell population as they are readily accessible in human blood, easy to culture and expand *in vitro *and are of direct relevance for infection [43]. Another disadvantage of *in vitro *stimulation with phosphoantigens may be the induction of inhibitory receptors on γδ T cells, which may modulate their response in disease state differentially than HC [44].

The activation of γδ T cells can be cytolytic or inflammatory by secretion of cytokines [45]. Previous studies pointed to a role of activated γδ T cells in BD [17,31,46]. The present study did not provide evidence for an increase of γδ T cells in active BD patients, although functional changes implicated by surface receptors are shown in BD.

A major limitation of our study is the assessment of γδ T cells function with limited stimulation in an *in vitro *culture system. Other PB cell subsets and cytokines/chemokines might influence the response of γδ T cells. With different methodology, such as single-cell experiments and measurement of mRNA expressions, role and function of γδ T cells in BD pathogenesis might be further clarified.

## Conclusions

In the present study no increase of γδ T cells was detected in active BD patients. However, based on surface receptors on γδ T cells, functional changes of these cells were implicated. When TCRVδ2^+ ^T cells were expanded and tested for the response to TCR and TLR stimulations, cytokine and chemokine secretions were found impaired in BD.

## Abbreviations

A-BD: active BD; BD: Behcet's disease; BrHPP: bromohydrin pyrophosphate; CRP: C-reactive protein; ESR: erythrocyte sedimentation rate; GM-CSF: granulocyte monocyte colony stimulating factor; HC: healthy control; IA-BD: inactive BD; IFN: interferon; IL: interleukin; IPP: isopentenyl pyrophosphate; mAb: monoclonal antibodies; MS: multiple sclerosis; PB: peripheral blood; PBMCs: peripheral blood mononuclear cells; PPD: purified protein derivative; RANTES: Regulated on Activation, Normal T Expressed and Secreted; TB: tuberculosis; TCR: T cell receptors; TNF: tumor necrosis factor.

## Competing interests

The authors declare that they have no competing interests.

## Authors' contributions

GP and EG participated in patient data collection, performed phenotyping and cell culture, interpreted and analyzed the data, and wrote the manuscript. BE and ZK participated in the design of the study, sample and data collection from the patient groups, interpretation of the data and manuscript preparation. AG, HD and GSD designed and coordinated the study, analyzed and interpreted the data, and wrote the manuscript. All authors read and approved the final manuscript for publication.

## Supplementary Material

Additional file 1 **Figure S1**. Induced GM-CSF, IFN-γ, IL-13, CCL4, CCL5 and TNF-α levels with TCR alone and with additional co-stimulation of TLR3 agonist are shown in the HC group (*n *= 20). Click here for file
