# Supplementary figures and images for: Expression of regulatory receptors on γδ T Cells and their cytokine production in Behcet's disease
Source: Arthritis Res Ther. 2013 Jan 21;15(1):R15. doi: 10.1186/ar4147 (PMC3672743; doi:10.1186/ar4147)

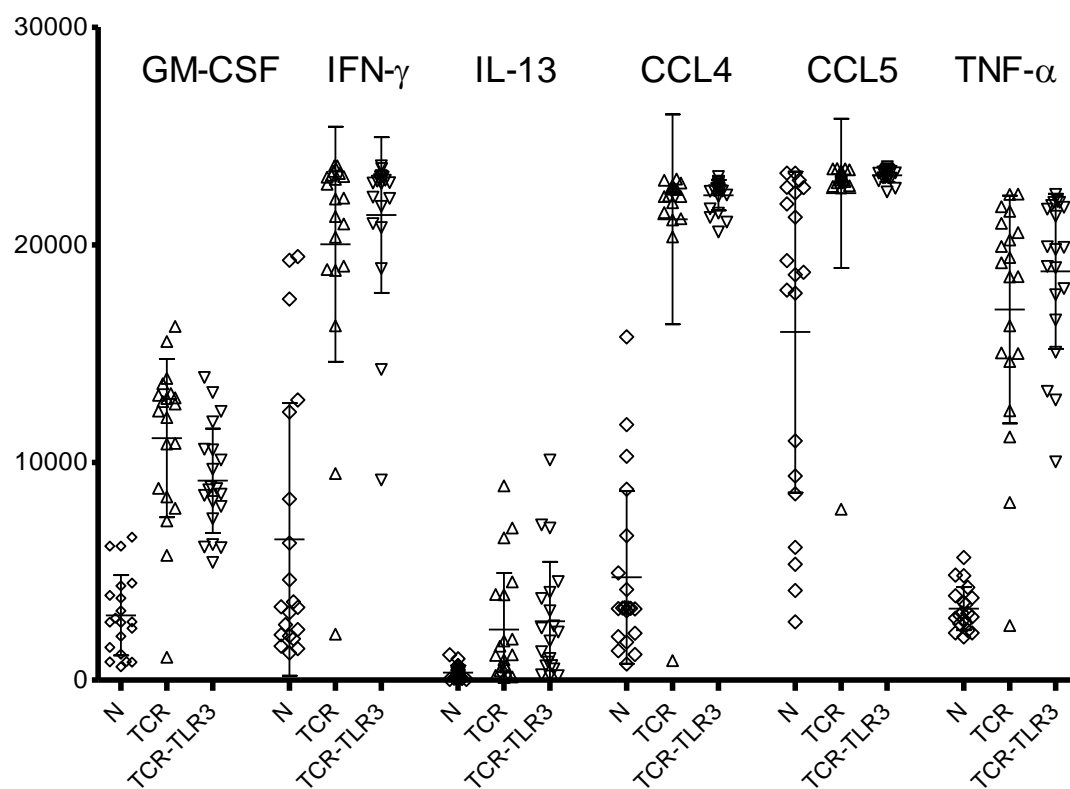

Supplement: Additional file 1 — Figure S1. Induced GM-CSF, IFN-γ, IL-13, CCL4, CCL5 and TNF-α levels with TCR alone and with additional co-stimulation of TLR3 agonist are shown in the HC group (n = 20). [file ar4147-S1.PDF]
